# Supplementary material for: Liquid-Infused Porous Film Self-Assembly for Superior Light-Transmitting and Anti-Adhesion
Source: Micromachines (Basel). 2022 Mar 30;13(4):540. doi: 10.3390/mi13040540 (PMC9025966; doi:10.3390/mi13040540)
Supplement: Supplementary file 1 [file micromachines-13-00540-s001.zip › micromachines-1646713-supplementary.pdf]

Supporting information

Liquid-Infused Porous Film Self-Assembly for Superior Light-Transmitting and Anti-Adhesion

Yang Liu, Xiaoyang Zhan, Yan Wang, Guang Liu, Deyuan Zhang, Liwen Zhang * and Huawei Chen *
School of Mechanical Engineering and Automation, Beihang University, Beijing 100191, China
*Correspondence: chenhw75@buaa.edu.cn; Tel.: +86-010-8233-9717; lwzhang@buaa.edu.cn

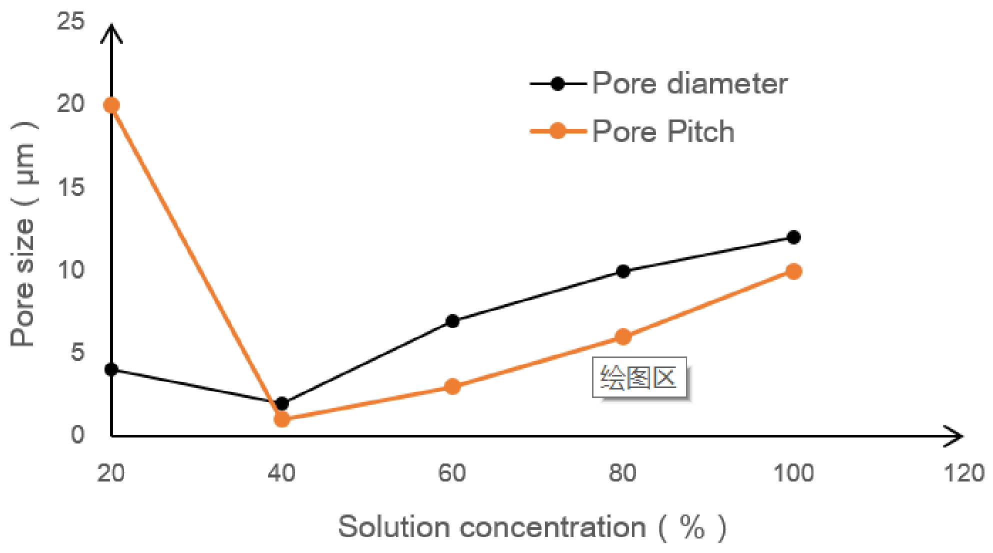

Figure S1. Plots of pore size versus solution concentration

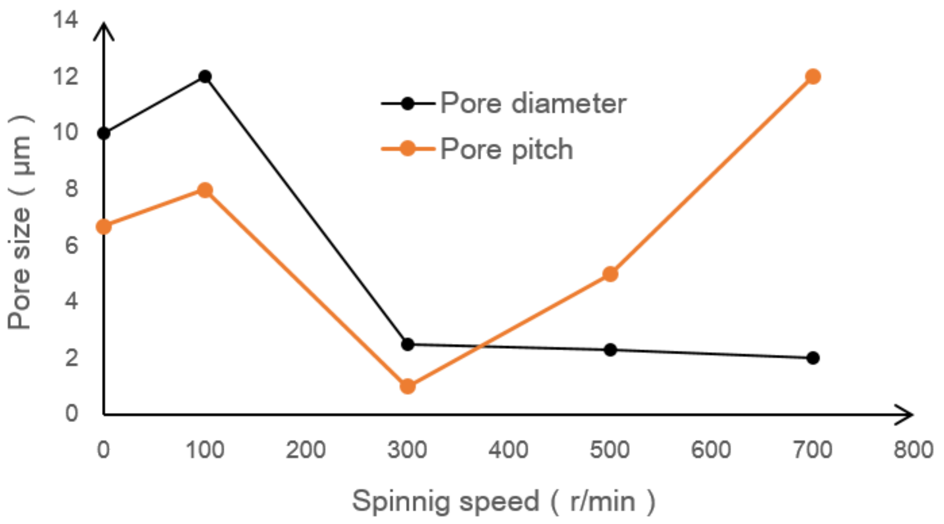

Figure S2. Plots of pore size versus spinning speed

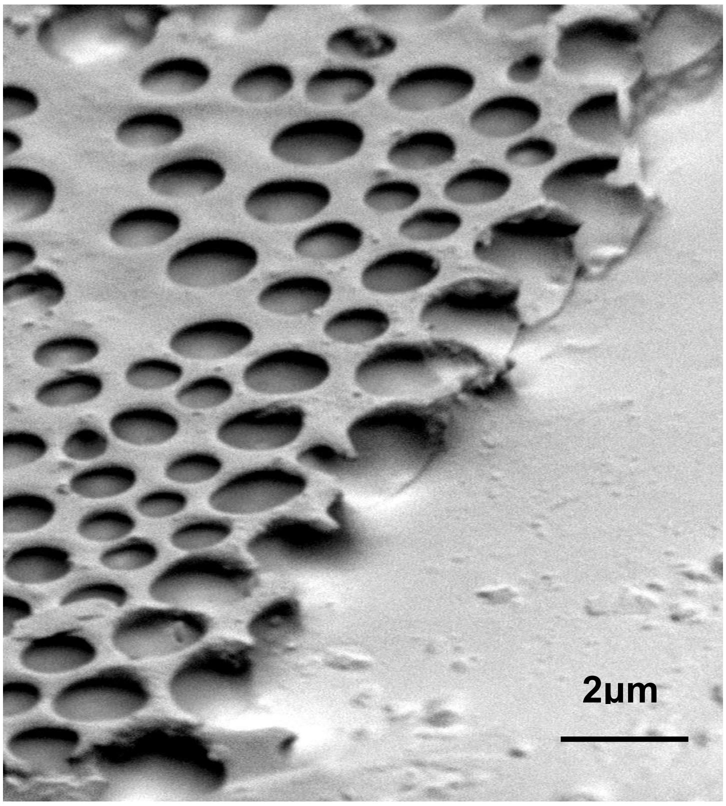

Figure S3. The side-view SEM photo of porous film

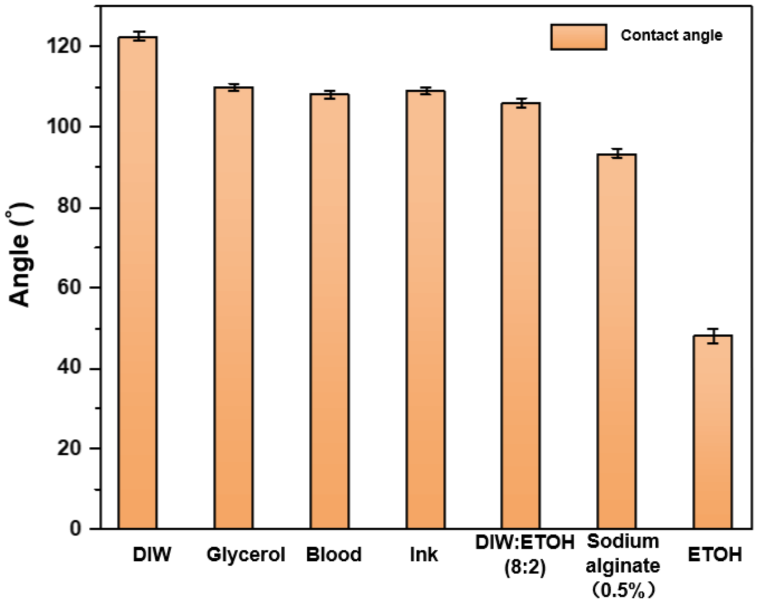

Figure S4. Contact angles of different droplets on the surface of a dry porous membrane
